# Supplementary material for: Heg1 and Ccm1/2 proteins control endocardial mechanosensitivity during zebrafish valvulogenesis
Source: eLife. 2018 Feb 1;7:e28939. doi: 10.7554/eLife.28939 (PMC5794256; doi:10.7554/eLife.28939)
Supplement: Transparent reporting form [file elife-28939-transrepform.docx]

***eLife’s* transparent reporting form**

We encourage authors to provide detailed information *within their submission* to facilitate the interpretation and replication of experiments. If you have any questions, please contact us: [editorial@elifesciences.org](mailto:editorial@elifesciences.org).

**Sample-size estimation**

- You should state whether an appropriate sample size was computed when the study was being designed
- You should state the statistical method of sample size computation and any required assumptions
- If no explicit power analysis was used, you should describe how you decided what sample (replicate) size (number) to use

Please outline where this information can be found within the submission (e.g., page numbers or figure legends), or explain why this information doesn’t apply to your submission:

Below, is a summary list that provides information on where the sample-size estimation for each experiment is presented:

Figure 1A-B and qRT-PCR experiments: informations can be found at page 30-31.

Experiment shown in Figure 1C-E: informations can be found at page 30

Experiment shown in Figure 2A: page 30-31

Experiment shown in Figure 2B-F: page 28-30

Experiment shown in Figure 3A-F: page 9 (main text), page 25-26

Experiment shown in Figure 3G-L: Figure Legend, page 15

Experiment shown in Figure 4A-B: more than 30 embryos analyzed by visual inspection, additional pictures are available

Experiment shown in Figure 4C-D’: page 11 (main text)

Experiment shown in Figure 1 suppl 1: more than 10 embryos analyzed per experimental condition

Experiment shown in Figure 1 suppl 2: Figure legend, page 17

Experiment shown in Figure 2 suppl 1: page 8 (main text)

Experiment shown in Figure 3 suppl 1: 82 rescued embryos analyzed

Experiment shown in Figure 3 suppl 2: page 27

Experiment shown in Figure 3 suppl 3: page 30-32

Experiment shown in Figure 3 suppl 4: Figure legend, page 18

Experiment shown in Figure 3 suppl 5: page 10 (main text), page 25-26

Experiment shown in Figure 4 suppl 1(A-D): more than 10 embryos analyzed per condition

**Replicates**

- You should report how often each experiment was performed
- You should include a definition of biological versus technical replication
- The data obtained should be provided and sufficient information should be provided to indicate the number of independent biological and/or technical replicates
- If you encountered any outliers, you should describe how these were handled
- Criteria for exclusion/inclusion of data should be clearly stated
- High-throughput sequence data should be uploaded before submission, with a private link for reviewers provided (these are available from both GEO and ArrayExpress)

Please outline where this information can be found within the submission (e.g., page numbers or figure legends), or explain why this information doesn’t apply to your submission:

For all RT-qPCR experiments, 3 to 4 biological replicates were performed (independent experiments using embryos from different clutches).

To determine the cycle threshold for each round of amplifications, we performed 3 technical replicates (details on page 30-31).

For each experiment reported, we performed at least 3 biological replicates, repeating the same experimental procedure in at least 3 different experimental sessions.

**Statistical reporting**

- Statistical analysis methods should be described and justified
- Raw data should be presented in figures whenever informative to do so (typically when N per group is less than 10)
- For each experiment, you should identify the statistical tests used, exact values of N, definitions of center, methods of multiple test correction, and dispersion and precision measures (e.g., mean, median, SD, SEM, confidence intervals; and, for the major substantive results, a measure of effect size (e.g., Pearson's r, Cohen's d)
- Report exact p-values wherever possible alongside the summary statistics and 95% confidence intervals. These should be reported for all key questions and not only when the p-value is less than 0.05.

For all RT-qPCR experiments, we used the ratio paired t test, because embryos used in WT versus treated conditions are siblings from the same egg clutch. Since we define the average fold-change values of the control as 1, we assessed whether the average ratio between the fold-change values of the treated samples and 1 was significantly different from 1 (additional information is provided on pages 30-32).

For experiments shown in figures 1E, 2E-F, 1 suppl 2C, 3 suppl 2, we used the unpaired t test to check whether the average values from two different populations that have an equal variance is significantly different (additional information is found on pages 30 for figure 1E and 1 suppl 2C, pages 28-30 for figure 2E-F, and pages 27 for figure 3 suppl 2)

For the experiments shown in figure 3A-F, we used a 2-way ANOVA test, to compare 3 different experimental conditions in three independent biological replicates.

Please outline where this information can be found within the submission (e.g., page numbers or figure legends), or explain why this information doesn’t apply to your submission:

(For large datasets, or papers with a very large number of statistical tests, you may upload a single table file with tests, Ns, etc., with reference to page numbers in the manuscript.)

**Additional data files (“source data”)**

- We encourage you to upload relevant additional data files, such as numerical data that are represented as a graph in a figure, or as a summary table
- Where provided, these should be in the most useful format, and they can be uploaded as “Source data” files linked to a main figure or table
- Include model definition files including the full list of parameters used
- Include code used for data analysis (e.g., R, MatLab)
- Avoid stating that data files are “available upon request”

Please indicate the figures or tables for which source data files have been provided:

This does not apply.
